# New Antioxidant Multilayer Packaging with Nanoselenium to Enhance the Shelf-Life of Market Food Products

**DOI:** 10.3390/nano8100837

**Published:** 2018-10-16

**Authors:** Paula Vera, Elena Canellas, Cristina Nerín

**Affiliations:** 1 Analytical Chemistry Department, GUIA Group, I3A, EINA, University of Zaragoza, Mª de Luna 3, 50018 Zaragoza, Spain; pvera@unizar.es (P.V.); elenac@unizar.es (E.C.); 2 Samtack Adhesivos Industriales, C/Cerámica, nº3, Pol. Ind. Magarola Sud, 08292 Esparraguera, Barcelona, Spain

**Keywords:** selenium nanoparticles, multilayer laminates, antioxidant packaging, TBARS

## Abstract

A flexible multilayer with selenium nanoparticles incorporated has been used to build an antioxidant packaging. The oxidation of hazelnuts, walnuts, and potato chips was tested at laboratory scale. Hexanal released by the nuts, fatty acids oxidation study, TBARS (thiobarbituric acid reactive substances), and tasting were compared to study the oxidation of foods packaged with this antioxidant packaging. Finally, TBARS method in combination with tasting were selected due to their simplicity and accuracy. It was found that hazelnuts packaged in nanoSe active bags released around 20% less malonaldehyde (MDA) than the blanks. In the case of the walnuts, the active ones released 25% less MDA than the blanks. As for potato chips, the improvement was around 22%. Finally, an industrial study was done. Cooked ham, chicken, and a ready-to-eat vegetable mixture seasoned with butter were industrially packaged with the new antioxidant material and improvements higher than 25% were obtained.

## 1. Introduction

Lipid oxidation is an important factor that limits the shelf-life of food. It reduces the nutritional value of lipids, some fat-soluble vitamins are lost because of its reaction with free radicals, some pigments disappear, and rancidity starts. This is one of the main causes of food product rejection by consumers, since it is associated with characteristic off-flavor due to the generation of volatile short-chain aldehydes and ketones, which are responsible for off-flavor, such as malonaldehydes (MDA) [1].

The development of active packaging provides an opportunity to extend the freshness of food products. Antioxidant packaging may act by absorbing the compounds that deteriorate the food, such as oxygen or free radicals. Oxidation process is a radical reaction initiated by the free radicals derived from oxygen, which are the primary free radicals. These free radicals are transferred to the lipid chains and their oxidation takes place. Previous works demonstrates that scavenging these primary free radicals is the best and the most efficient way to protect food against oxidation [2,3,4,5,6,7,8]. The antioxidant properties of selenium nanoparticles (SeNPs) are well known [9,10,11,12], and based on these concepts a new multilayer material containing nanoSe was built and optimized [13]. This new antioxidant polymer was optimized and studied at lab scale using oxidizable model compounds. However, tests with real food at both lab scale and industrial scale were not carried out. The scientific literature shows many approaches of antioxidant packaging materials, but most of them fail when trying to apply them at industrial level in a packaging line. Machinability of the new active materials, as well as stability, off-odor, color, and other physical characteristics, besides the antioxidant performance, are the main reasons why these new approaches are not common in the food market. Industrial scale up is always a big challenge and often the positive results from laboratory tests turn into a different panorama, where the gain of the new material is not acceptable. In this work, an antioxidant packaging based on selenium nanoparticles incorporated to a flexible multilayer was tested with food susceptible to rancidity. Then, in addition to the mentioned difficulties, Se nanoparticles had to be produced and handled at industrial level and incorporated in a homogeneous and reliable manner into the packaging material. The shape, size, and distribution in the packaging, as well as stability, had to be under control and the resulting material should be characterized and tested in real situations. The antioxidant capacity of the laminates with SeNPs incorporated have been previously demonstrated by Vera et al. at laboratory scale. It was confirmed that SeNPs are able to trap the primary free radicals derived from oxygen [13]. With this information, the new multilayer material containing nanoSe has recently been evaluated and it received a positive opinion from EFSA (European Food Safety Authority). The absence of migration of nanoparticles, together with the efficiency as an antioxidant at lab scale were the guarantee of food safety. Thus, it can be scaled up and launched into the market, as one of the few active materials in the EU market.

The first goal is to monitor the oxidation reaction of packaged food during its shelf life. One of the strategies to evaluate this is through a sensory panel. This methodology is very useful when the antioxidant packaging is designed to increase the time the consumer considers the food acceptable. Nevertheless, this methodology depends on the people that form the panel, and it is less reproducible than an instrumental method. Thus, it is important to combine this panel with analytical techniques that provide accurate measurements, such as the determination of the fatty acid composition of the food [14,15,16]. The method consists of derivatizing the fatty acids to their glyceryl esters, which will be analyzed either by GC-FID or GC-MS. This way, a quantitative value of each fatty acid before and after the oxidation will be obtained, and consequently the amount of fatty acids that disappear because of the oxidation will be available. Other techniques are based on the determination of the volatile compounds responsible for the unpleasant odors and in some cases propanal, pentanal, hexanal, 2-hexenal, 3-hexenal, or 2,4-heptadienal [17,18] release were also monitored to confirm rancidity. A common methodology in food is the thiobarbituric acid reactive substances (TBARS) assay. This method is based on the determination of malondialdehyde (MDA), which is a low-molecular-weight end product usually formed via the decomposition of certain primary and secondary lipid peroxidation products [19,20]. TBARS technique has been used in many oxidation studies of food [21]. To have an objective evaluation of the industrial production of this industrial nanoSe antioxidant packaging, all these methodologies were tested to demonstrate the efficiency of the new nanoSe active packaging on packaged food. 

The main targets for oxidation in the lipids are the polyunsaturated fatty acids, which are vulnerable to the action of free radicals. This is due to the presence of double bonds that weaken the C-H bonds [22,23]. The hydrolysis and/or autoxidation of fats provide short-chain aldehydes and ketones responsible for rancidity, and rancidity is associated with the characteristics off-flavor and odor [1]. Nuts have a high content of unsaturated fatty acids [24]. Owing to that, walnuts and hazelnuts were the first foods selected for this study. According to the literature, walnuts have a content of monounsaturated fatty acids (MUFA) of around 9 g/100 g and a content of polyunsaturated fatty acids (PUFA) of around 48 g/100 g. Hazelnuts have a MUFA value of approximately 46 g/100 g and PUFA of approximately 8 g/100 g [25]. Therefore, they are good candidates to study their lipid oxidation and rancidity [26], and consequently nuts and walnuts were the first target of this study. Then, several foods susceptible to rancidity, packaged by different companies and packaging lines, have been analyzed and the results are shown and discussed.

## 2. Materials and Methods

### 2.1. Reagents

Dichloromethane, cyclohexane, sodium hydroxide, boron trifluoride, sodium chloride, trichloroacetic acid, thiobarbituric acid, and 1,1,3,3-tetraethoxypropane were purchased from Sigma-Aldrich (Sigma-Aldrich-Merck, Madrid, Spain). Methanol and heptane were supplied by Scharlau Chemie S.A (Scharlau, Sentmenat, Spain). Solid phase microextraction fibers were supplied by Supelco (Bellefonte, PA, USA).

### 2.2. Laminates

**Figure 1:**
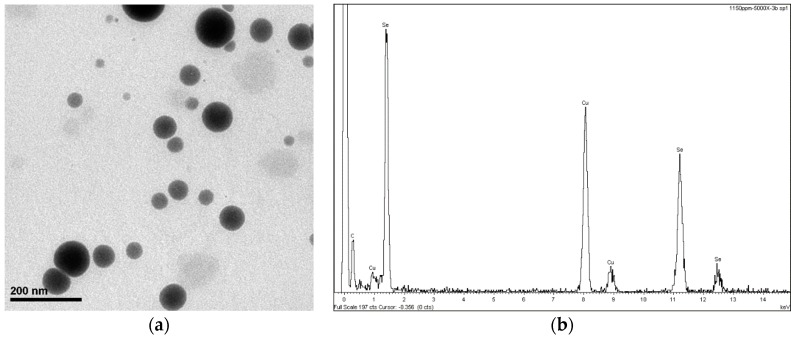
(**a**) SEM of 1500 ppm solution of nanoSe and (**b**) EDX analysis of SeNPs. Samples on Cu grid.

Laminates made with selenium nanoparticles were prepared. Firstly, selenium nanoparticles were synthesized using a solution-phase approach based on the reduction of selenite with ascorbic acid in the presence of 7% of concentration of 2,4,7,9-tetramethyl-5decyne-4,7-diol ethoxylate as stabilizer agent (at pH 6.5). The final concentration of selenium nanoparticles in the solution were 1000 ppm. SeNPs between 50 and 70 nm were obtained. Figure 1 shows the SEM analysis, where the size and identification of SeNPs in the solution can be seen. The detailed description of the synthesis can be read in Vera et al. [13].

This nanoselenium solution was incorporated at 10% (*w*/*w*) into a water-based adhesive dispersion. The formula of adhesive was provided under a confidential agreement and cannot be disclosed here.

Using the adhesive, the laminate structure [substrate 1-adhesive-substrate 2] was manufactured first at laboratory scale and later at industrial scale. Industrial conditions were optimized as well to provide a colorless multilayer, where nanoSe was homogeneously distributed. At laboratory scale, the laminates were prepared using an extender machine K control coater, RK printcoat instruments (RK PrintCoat Instruments Ltd, Litlington, UK) on a 20 × 30 cm of substrate 1 polyethylene terephthalate (PET, 12 μm), forming a uniform layer. The grammage of adhesive placed was 3 g/m^2^, which was gravimetrically measured. Afterwards, a second 20 × 30 cm of 60 μm thickness film of low density polyethylene (LDPE) was placed on top of it, and then the laminate was pressed at 80 °C at speed number 4 in a BiO 330 model Laminator (Laminator-BIO 330, Korea)

Blank laminates were also prepared as follows: PET (12 μm)/adhesive/PE (60 μm) (laminate B) using the same formula without nanoSe. 

### 2.3. Food Samples and Packagings

Hazelnuts, walnuts, and potato chips were bought in bulk. Fresh hazelnuts and walnuts without the peel were used. Negret hazelnut and Hartley walnut variety, additive free, were selected. Fresh fried potato chips with the following composition: Sunflower oil 80% and olive oil 20% were chosen for the study, assuming that they were prone to oxidation. In the studies made at laboratory scale, 5 × 2.5 cm^2^ thermosealed bags were made with the laminates using a thermal sealer. An LDPE layer was inside the bag in contact with the food. Nuts and potato chips were ground to increase the surface in contact with the packaging, and to increase the oxidation effect to see differences in the oxidation of blanks and actives.

Industrial samples were provided by several companies interested in the study. Their names are confidential. Breaded Wiltshire cured ham (British stile ham) was studied. No antioxidants were added to the ham. Ready-to-eat vegetable mixture seasoned with butter consisting of sweet corn, chopped broccoli, and chopped carrot were studied. Fresh raw chicken breast was also packaged by another food company. In all cases, their own industrial packaging line and their own format (shape and size) for the selected food were used, without intervention or participation of people involved in the nanoSe project and development.

### 2.4. Monitoring the Hexanal 

Hexanal analysis was done by headspace-solid phase microextraction (HS-SPME) coupled to a gas chromatography mass spectrometry (GC-MS). The SPME selected was Divinylbenzene/Carboxen/Polydimethylsiloxane (DVB/CAR/PDMS). The fiber was introduced into the bags made with the laminates, which contained the nuts. The extraction time was 15 min at room temperature and the desorption was done at 250 °C in the GC-MS. The equipment used was an Agilent 6890N gas chromatograph with a mass spectrometer MS 5975B detector. All of them were from Agilent Technologies (Madrid, Spain). 

The capillary column used was a HP-5MS (30 m × 0.25 µm × 250 µm) from Agilent Technologies (Madrid, Spain). The oven program was as follows: 40 °C for 2 min, with a rate of 10 °C/min up to 300 °C, maintained for 2 min. The injection type was splitless and the helium flow was 1 mL/min. The mass detector was set at SCAN mode (in the range *m*/*z* 45–350) for the identification of the compounds, and SIM mode for the quantification of the compounds in the migration extracts.

### 2.5. Fatty Acids Oxidation Study

In the fatty acids oxidation study, a derivatization of the fatty acids was performed, where 10 g of grounded nuts were added to a recipient and then 20 mL of DCM:cyclohexane (1:1) were added. The two phases were separated. The liquid phase was transferred to another recipient and dried at room temperature. Then, 4 mL of NaOH 0.5 M were added to a 100 mg of this extract. This mixture was kept boiling for 10 min. Then, 5 mL of boron trifluoride 14% in methanol were added and the mixture was kept boiling for 2 min. Finally, 4 mL of heptane were added and the mixture was kept boiling for 1 min. The mixture was allowed to cool for 2 min. Subsequently, 15 mL of NaCl were added and the mixture was shaken with a Vortex®. 

The organic phase was analyzed by GC-MS. The equipment used was a CTC Analytics CombiPal autosampler coupled to an Agilent 6890N gas chromatograph with a mass spectrometer MS 5975B detector. All of them were from Agilent Technologies (Madrid, Spain). 

The capillary column used was a HP-5MS (30 m × 0.25 µm × 250 µm) from Agilent Technologies (Madrid, Spain). The oven program was as follows: 40 °C for 2 min, with a rate of 10 °C/min up to 300 °C, maintained for 2 min. The injection type was splitless and the helium flow was 1 mL/min. The mass detector was set at SCAN mode (in the range *m*/*z* 45–350) for the identification of the compounds, and SIM mode for the quantification of the compounds in the migration extracts.

### 2.6. TBARS (Thiobarbituric Acid Reactive Substances)

The oxidation of lipids was checked using the thiobarbituric acid reactive substances (TBARS) method. The assay was carried out following the method developed by Pfalzgraf et al. [23]. Briefly, 10 g of food were mixed with 20 mL of aqueous solution of trichloroacetic acid at a concentration of 10 μg/g, and then homogenized with an Ultra-Turrax at 18,000 rpm till uniform slurry was obtained. The supernatant was filtered using qualitative paper filter. Then, 2 mL of the filtrate were mixed with 2 mL of aqueous solution of thiobarbituric acid at a concentration 20 mM. The mixture was kept in a thermostatic bath at 97 °C during 20 min. The absorbance of the solution was measured at 532 nm against the blank sample (instead of filtered aliquot, 2 mL of trichloroacetic acid solution was used to prepare the blank sample). The results were expressed in equivalent concentration of malondialdehyde (MDA; mg of malondialdehyde/kg of meat). The malondialdehyde solution was prepared from 1,1,3,3-tetraethoxypropane dissolved in 1N aqueous hydrochloric acid for the calibration curve.

### 2.7. Tasting

The tasting was done by an expert panel composed of 10 tasters (8 women and 2 men). They were tasters trained for 5 years in this kind of tasting with antioxidant packaging and using different foodstuffs and different antioxidant agents in the packaging. They were also experts on active packaging materials tastings. They scored four parameters from 1 (worse) to 5 (better): appearance, aroma, taste, and rancidity. It was always a blind tasting and the data were processed by Statistical Package for the Social Sciences (SPSS) software.

## 3. Results

The antioxidant packaging was firstly evaluated using the methodology described by Pezo et al. [3], based on the exposure of the packaging material to an atmosphere enriched in free radicals. This method, where the free radical scavenging system is applied directly on the film, without requiring a previous extraction of the polymer, allows the evaluation of real antioxidant performance of the packaging. This method was necessary, as Vera et al. [13] demonstrated that the free radical diphenylpicrylhydrazyl (DPPH) was too big to cross the LDPE layer and arrive at the adhesive where the antioxidants, the SeNPs, are placed. Thus, the antioxidant performance can be considered as the actual one corresponding to the film. The material under study, containing SeNPs, was not previously tested on food.

As the aim of this work was to demonstrate the efficiency of this antioxidant packaging to protect the food versus oxidation processes, two series of tests were designed, the first series at laboratory scale and the second one at industrial level. 

Several methodologies were applied and compared to objectively measure the oxidation of fats. 

### 3.1. Monitorization of Hexanal Released by the Nuts

Hexanal is the main product of linoleic acid oxidation. Therefore, it is an indicator of lipid oxidation, which has been used as a good marker to monitor many foods: Potato crisps [27], Rapeseed oil [28], Iberian dry-cured loin (Muriel et al. 2007) meat and meat derivatives [29]. Nonanal and hexanal were measured in olive oil [30] and also in nuts [31]. 

The SPME fiber selected was Carboxen/Polydimethylsiloxane (CAR/PDMS) with 75 µm thickness, since according Pastorelli et al. [32] it had the highest sensitivity for this compound. The limit of detection for hexanal was 5 ng mL^−1^.

In our case, hexanal did not result in a good marker. Since hexanal is a small molecule, it was able to pass through the laminate that formed the bags. Hexanal was measured after 9, 27, 63, and 105 days of storage. It was observed that the amount of hexanal was gradually decreasing throughout the study, probably due to its permeation throughout the multilayer. Therefore, the hexanal value could not be considered as reliable in this case. This also demonstrates that the presence of nanoSe in the multilayer does not enhance the barrier properties of the multilayer versus organic vapors, such as aldehydes. It is the first time that this permeation of hexanal in the presence of nanoSe in a multilayer is described. 

Subsequently, this methodology for monitoring the oxidation versus time was discarded. 

### 3.2. Fatty Acids Oxidation Study

It has been demonstrated that the fatty acid composition of oils is a strong indicative factor that could predict the oxidative state [14,15,16]. Owing to this, the determination of fatty acids was selected here to study their oxidation rate.

Derivatization was the selected methodology for the fatty acid analysis by GC, since the use of ester derivatives is recommended. The methyl ester derivatives are much more volatile than the corresponding fatty acids. Moreover, they are much less polar, avoiding peak tailing and peak asymmetry on GC [33]. 

Methyl palmitate, methyl linoleate, methyl oleate, and methyl stearate were the esters determined in this work, since palmitic acid, linoleic acid, oleic acid, and stearic acid are the most abundant fatty acids in hazelnuts and walnuts [34].

Table 1 shows the amount of these fatty acids expressed as ng/g hazelnuts and as ng/g walnuts in the bags made with laminate A (made with adhesive alone) and laminate B (made with adhesive + 10% of nanoSe). Fatty acid values were around 50% higher in the bags made with laminate B, which contained nanoSe at both day 21 and day 42, respectively. The highest differences were found for walnuts. An improvement on the amount of fatty acids from 50 to 66% at day 42 was found in the active bag. It can be seen that 11.3 ng/g of methyl oleate were found in the bag made with laminate B and 3.88 ng/g were found in the bag made with laminate A. In the case of hazelnuts, the differences on the fatty acid amount between the active bag and the blank ranged from 24 to 53% at day 42. Therefore, these results demonstrate the antioxidant effect on food and the consequent shelf life extension of the packaged nuts, since pristine fatty acids remain for much more time in nuts.

### 3.3. TBARS (Thiobarbituric Acid Reactive Substances)

Unsaturated fatty acids are oxidized to form odor-free, tasteless hydroperoxides. Then, they are decomposed to flavorful secondary oxidation products, which are mainly aldehydes, such as hexanal, 4-hydroxynonenal (HNE), and malondialdehyde (MDA) [35]. The most common method to determine MDA in foods is the spectrophotometric measurement of the colored adduct of MDA with 2-thiobarbituric acid (TBA). This methodology is not specific, but it has been reported to be a more accurate and sensitive parameter in assessing the oxidative deterioration than other methods [36].

**Figure 2:**
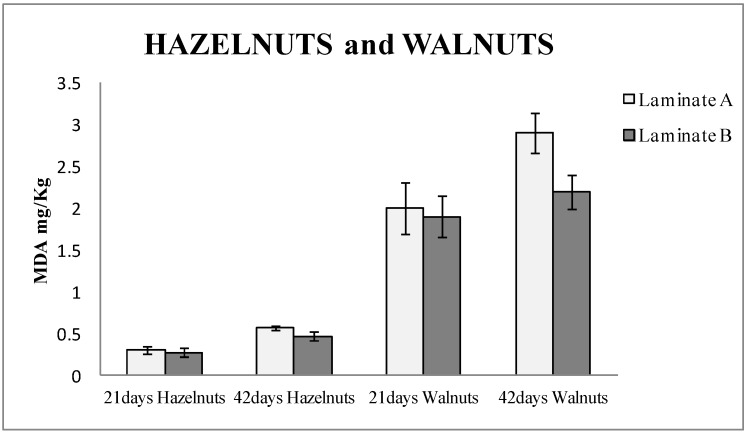
Thiobarbituric acid reactive substances (TBARS) results expressed as mg of malonaldehyde (MDA) per kg of hazelnuts and walnuts packaged in laminate A (control) and laminate B (actives with nanoselenium particles) for 21 and 41 days, respectively.

Figure 2 shows the TBARS results of hazelnuts and walnuts packaged for 21 and 42 days, respectively, in both types of laminates, control and active. It can be observed that the hazelnuts packaged in active bags (made with laminate B) released around 10% less MDA than the blanks (laminate A) after 21 days of being packaged, and up to 20% when they were measured after 42 days. In the case of the walnuts study, the bags made with laminate B (active) released 5% less MDA than the blanks (laminate A) after 21 days and around 25% less after 42 days. The values of MDA were higher in walnuts than in hazelnuts. This can be attributed to the fact that walnuts contain a higher amount of PUFA [25], which are more vulnerable to oxidation. Student T-test was performed, and the results were 0.005 for hazelnuts and 0.016 for walnuts. Thus, it could be confirmed that the results with laminate A and B had significant differences with a 95% confidence level. 

The results also demonstrated that the active packaging based on nanoSe prevents the oxidation of nuts and significantly extends the shelf life of the nuts.

### 3.4. Sensory Evaluation of Packaged Nuts

The tasting was done by an expert panel composed of 10 tasters. They scored four parameters from 1 (worse) to 5 (better): appearance, aroma, taste, and rancidity after 42 days of storage of the bags. 

**Figure 3:**
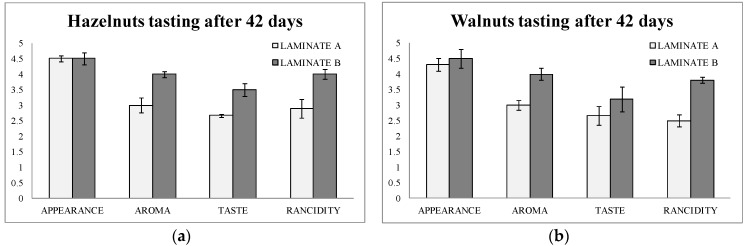
(**a**) Sensory evaluations of hazelnuts packaged in laminate A (control) and laminate B (actives with nanoselenium particles) after 42 days; (**b**) Sensory evaluations of walnuts packaged in laminate A (control) and laminate B (actives with nanoselenium particles) after 42 days.

Figure 3a,b show the results of the tasting. It can be observed that aroma, flavor, and rancidity were scored at higher values in both hazelnuts and walnuts in laminate B (active) than in laminate A (blank). Rancidity obtained a difference of score of one point between hazelnuts packaged in active bags after 42 days of storage. Moreover, in walnuts the difference was even higher for rancidity (1.3 points of difference).

This data were of great importance, since the consumer will judge the usefulness of the new active packaging. In addition to this, it can be underlined here that the tasting data matched with the analytical data found above by the two methods used.

### 3.5. Industrial Tests 

Once different methodologies to study the oxidation were tested at laboratory scale in nuts, the active packaging was produced at industrial scale and used in other foods. TBARS method in combination with tasting (for ready-to-eat foods) were the methodologies selected for this work. The analysis of fatty acids was discarded, since TBARS offered sufficient results to compare active and blank packaged food, and TBARS was also simple and quicker.

To begin with, a laboratory scale study was done with the industrial multilayer to conduct an additional study of the active packaging with real food prior the industrial scale. The industrial active multilayer was prepared following the same steps as in the laboratory. Firstly, nanoparticles were prepared and incorporated into the adhesive. This active adhesive was then applied between the two plastic films to form the multilayer. This preliminary test was designed to save money and food coming from a large test at industrial scale in a food company. Once the antioxidant properties of the industrial multilayer were confirmed, the industrial tests with different food produce were carried out. In the preliminary study, potato chips, which are susceptible to oxidation, were selected. Once the results of this study were obtained, industrial trials were prepared with ham, vegetables seasoned with butter, and fresh chicken. 

### 3.6. Potato Chips (Laboratory Scale Study)

Potato chips were selected for this study as they have a high amount of unsaturated fats, since they were fried in olive oil, according to the information provided by the suppliers. This kind of product is susceptible to rancidity, and it turns into a perfect target for the antioxidant packaging study.

These potato chips did not contain preservatives or antioxidants, as was mentioned in the experimental section. The time for the study was established according to the shelf-life of the product. The TBARS study and the tasting were done 21 days after the packaging.

**Figure 4:**
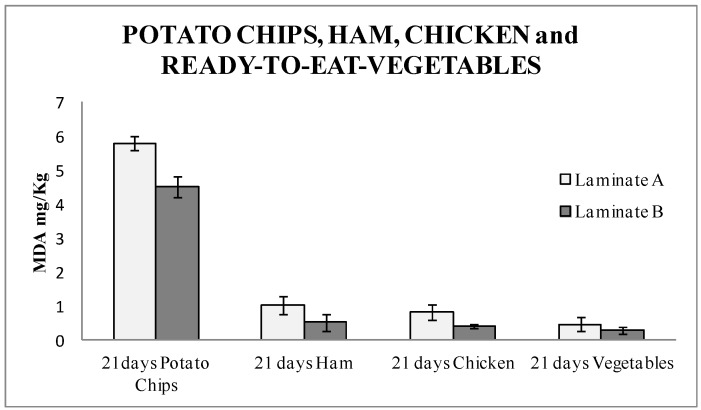
TBARS results expressed as mg of MDA per kg of potato chips, ham, chicken, and ready-to-eat-vegetables packaged in laminate A (control) and laminate B (actives with nanoselenium particles) for 21 days.

The TBARS results (Figure 4) showed a high amount of malonaldehyde. This result was due to the high amount of fat contained in the product. A significant difference of 22% was found between the active laminates and the blanks. This data demonstrated that potato chips were protected from oxidation in this packaging, and therefore this antioxidant packaging could be a good target for its implementation in the market. Student T-test was performed, and the result was 0.045. It was confirmed that the results with laminate A and B were statistically different with a 95% confidence level. 

**Figure 5:**
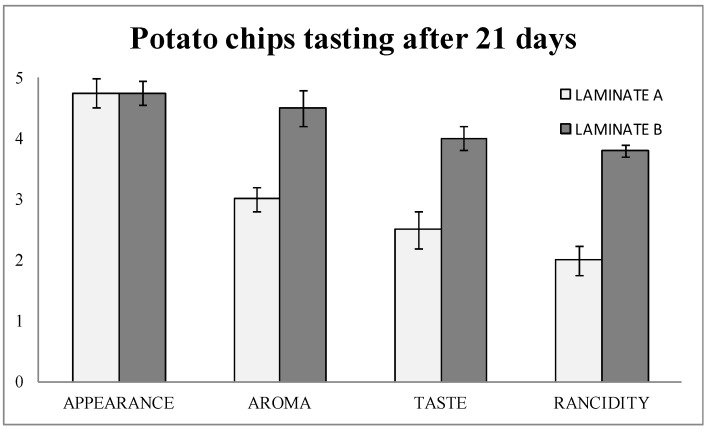
Sensory evaluations of potato chips packaged in laminate A (control) and laminate B (actives with nanoselenium particles) after 21 days.

The tasting results (Figure 5) showed that the detection of rancidity was almost two out of five points higher in laminate B (blank) than in laminate A (with nanoSe). Therefore, aroma and taste were consequently better in laminate A (nanoSe).

### 3.7. Industrial Study: Cooked Ham, Chicken and Ready-to-Eat Vegetables Dressed with Butter

Cooked ham, chicken, and ready-to-eat vegetable mixture seasoned with butter were the foods studied in the industrial trial, since producers of these kind of foods expressed their interest in the antioxidant packaging for their products. A large series of each food was packaged in their industrial packaging lines, using the same procedure, packaging speed, and system as the conventional multilayer. Blank and active samples were also packaged in each case and the same procedures above mentioned and optimized, were applied to monitor the behavior of the packaged food. Half the amount of the tested food was evaluated by the food companies and half by the research team at the University. All the results arrived at the same conclusions about the active nanoSe material. Drawbacks related to the packaging lines were not found, as the material behaved the same as the conventional one, because the active agent was in the middle of the multilayer, in sandwich mode, and consequently it did not affect the performance of the plastic.

Figure 4 shows the TBARS results for all of them packaged for 21 days. In the four types of foods under study, a significantly lower amount of MDA, around 50% for ham, released in the food packaged with the nanoSe were found. Therefore, this means a considerable improvement in the quality of the market produce, since less rancid products are released. This also turns into an extended shelf-life of food produce and demonstrates that the active packaging with nanoSe is a successful option. This is the first time that an industrial active multilayer containing Se nanoparticles as an antioxidant agent was studied. This study opens the door for further active materials, where nanoparticles can be introduced in a skilled manner without affecting the behavior of the material, whilst harnessing the properties and advantages of the packaging materials. Student T-test was performed, and the result was 0.04 for ham, 0.01 for chicken, and 0.06 for vegetables, respectively. The results with laminate A and B were significantly different with a 95% confidence level, for ham and chicken. Vegetables packed with laminate A and B were not significantly different. 

## 4. Conclusions

This works demonstrated that active packaging based on selenium nanoparticles prevents the oxidation of real food and enhances and extends its shelf-life. The presence of nanoSe in the multilayer did not increase the barrier properties for hexanal and aldehydes, but its performance as a strong free radical scavenger was demonstrated. Several industrial lines and food companies tested the material for a wide series of food, and in all cases considerable improvement of food protection versus oxidation was found. This is the first time that the efficiency of an active antioxidant material was demonstrated at industrial scale. In addition, it was demonstrated that the combination of the TBARS method with tasting (for ready-to-eat foods) were the most suitable methodology to evaluate the efficiency of the antioxidant packaging containing nanoSe. This study opens the door to new developments of active materials containing nanoparticles in which the nanoparticles are not in direct contact with the food, but act on food protection from the packaging. 

## Figures and Tables

**Table 1:** Concentration of fatty acids expressed as ng/g of hazelnuts and walnuts obtained for the laminates A (control, without nanoselenium particles) and laminates B (with nanoselenium particles) expressed as ng/g _food_.

	Hazelnuts		Walnuts	
	Laminate A	Laminate B	Laminate A	Laminate B
**Methyl Palmitate**				
Day 0	28.6 ± 3.4	28.6 ± 3.3	38.89 ± 3.0	38.89 ± 4.1
Day 21	8.04 ± 0.90	16.4 ± 1.51	12.1 ± 1.6	21.5 ± 1.9
Day 42	4.92 ± 0.21	9.72 ± 1.45	5.17 ± 0.42	10.3 ± 0.22
**Methyl Linolelate**				
Day 0	39.7 ± 7.4	39.7 ± 6.4	167 ± 10	167 ± 8
Day 21	12.9 ± 1.9	25.5 ± 3.6	35.7 ± 3.9	64.8 ± 8.4
Day 42	6.86 ± 0.91	10.9 ± 2.1	12.5 ± 1.6	21.2 ± 1.9
**Methyl Oleate**				
Day 0	58.4 ± 7.9	58.4 ± 8.1	66.6 ± 8.3	66.6 ± 8.0
Day 21	23.1 ± 3.1	37.3 ± 5.4	22.5 ± 1.4	39.8 ± 4.5
Day 42	14.4 ± 1.3	24.3 ± 1.5	5.22 ± 0.54	11.3 ± 1.7
**Methyl Stearate**				
Day 0	3.52 ± 0.33	3.52 ± 0.19	8.59 ± 0.99	8.59 ± 1.22
Day 21	3.32 ± 0.33	3.52 ± 0.35	4.57 ± 0.62	7.08 ± 0.77
Day 42	2.25 ± 0.22	2.34 ± 0.25	2.23 ± 0.29	4.45 ± 0.38
